# Solid‐State Nuclear Magnetic Resonance Investigations of the Lithium‐ and Sodium‐Storage Mechanisms of Pyrolytic Phosphorus‐Carbon Composites

**DOI:** 10.1002/cssc.202500103

**Published:** 2025-04-23

**Authors:** Cassius Clark, Christopher A. O’Keefe, Dominic S. Wright, Clare P. Grey

**Affiliations:** ^1^ Yusuf Hamied Department of Chemistry University of Cambridge Lensfield Road Cambridge CB21EW UK; ^2^ Cambridge Graphene Centre University of Cambridge 9 JJ Thompson Avenue Cambridge CB30FA UK; ^3^ The Faraday Institution Quad One, Harwell, Science and Innovation Campus Didcot OX11 0RA UK

**Keywords:** batteries, carbon, doping, graphites, nuclear magnetic resonance spectroscopy, phosphorus, sodium

## Abstract

Phosphorus‐doped carbons provide a balance between the electrochemical stability of graphitic lattices and the high energy density of phosphorus materials when used in lithium and sodium‐ion batteries. Herein, a comprehensive ex situ ^31^P, ^7^Li, and ^23^Na solid‐state nuclear magnetic resonance analysis of the intercalation mechanism of novel, stable, dual‐phase phosphorus‐doped, and phosphorus‐encapsulated turbostratic graphite microspheres is presented. Results indicate that lithium intercalation occurs through the formation of Li_3_P from white phosphorus trapped within the graphitic layers, with the involvement of lithiated phosphorus atoms within the graphitic lattice. A dual‐lithiated carbon doped‐phosphorus environment is tentatively proposed at low voltages. Sodiation occurs through a similar mechanism; however, no evidence of a dual‐sodiated doped‐phosphorus environment is observed. Upon removal of ions, carbon‐encapsulated phosphorus with a local structure similar to red phosphorus forms, which subsequently allows effective reversible ion storage.

## Graphical abstract

Phosphorus–carbon composites as battery electrode materials benefit from the high capacity of phosphorus and the stability of carbon. Understanding the ion‐storage mechanisms in these materials is crucial for their development. An analysis of the lithiation and sodiation mechanisms of a phosphorus–carbon composite is undertaken here utilizing ex situ solid‐state nuclear magnetic resonance, allowing a mechanistic insight into the multiple reactions that occur in these systems.

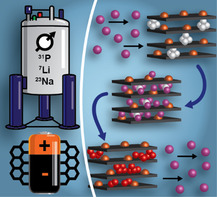

## 1 Introduction

Lithium‐ion batteries (LIBs) have remained the dominant electrical energy storage system, since 1991 when Sony released the first commercial LIB, owing to their high capacity and specific energy.^[^ ^1^ ^]^ However, the rising cost of lithium, which has increased by 738% since 2021,^[^ ^2^ ^]^ in part driven by its relatively low natural abundance, has motivated the development of improved battery systems with greater specific capacity coupled with an escalating interest in alternative “beyond‐Li” systems. In one example, the sodium‐ion battery, a series of new high‐capacity anodes has been developed that can reversibly store the significantly more abundant Na.^[^ ^3^, ^4^ ^]^

In conventional LIBs, the anode material is graphite, allowing the reversible intercalation of Li to form LiC_6_, giving a theoretical capacity of 372 mAh g^−1^.^[^ ^4^, ^5^ ^]^ By contrast, Na‐graphite intercalation compounds cannot be formed,^[^ ^6^ ^]^ unless a solvent is co‐intercalated.^[^ ^7^, ^8^, ^9^ ^]^ By contrast, the various allotropes of elemental phosphorus show promise as next‐generation anode materials, undergoing a three‐electron reaction to give a theoretical capacity of 2596 mAh g^−1^.^[^ ^10^ ^]^ However, the conversion reaction of elemental phosphorus to Li_3_P or Na_3_P results in a volume expansion of 216% and 391%, respectively. This can result in fracturing, electrical disconnection, and subsequent deactivation of active anode material. Consumption of electrolyte may then occur through side reactions on newly exposed surfaces.^[^ ^11^, ^12^ ^]^

Red phosphorus (RP) is an amorphous, air‐stable, and economical allotrope. However, its low electrical conductivity limits its usefulness as an electrode.^[^ ^13^ ^]^ To overcome this, composites of RP with carbon structures have been investigated, with varying results.^[^ ^13^, ^14^, ^15^, ^16^ ^]^ The use of conductive black phosphorus (BP) has been established in lithium and sodium cells.^[^ ^12^, ^17^, ^18^ ^]^ BP is the most stable form of phosphorus, comprising a layered structure of phosphorene sheets consisting of puckered P_6_ hexagonal rings.^[^ ^19^, ^20^, ^21^ ^]^ Formation of BP is not trivial and was originally synthesized in 1914 by heating white phosphorus to elevated temperatures and pressures, a technique still used in modern synthesis.^[^ ^17^, ^21^, ^22^, ^23^ ^]^ Other modern synthetic techniques utilizing ball milling or vapor deposition have been developed to produce varying quantities and levels of crystallinity of BP.^[^ ^18^, ^22^, ^24^, ^25^ ^]^ A comprehensive review of the synthesis of BP was recently published by Zeng et al.^[^ ^22^ ^]^ A first‐principles structure prediction study combined with nuclear magnetic resonance (NMR) calculations by Mayo et al. gave useful insight into the lithiation/sodiation processes of BP, identifying a number of important Li/Na_*x*_P compounds formed during cycling, the expected NMR chemical shifts, and the voltages for their formation.^[^ ^12^ ^]^ These results were consistent with a study by Marbella et al. on Na‐ion systems, which identified the voltages of (de)sodiation and the ^31^P and ^23^Na chemical shifts of the Na_*x*_P compounds formed during cycling.

The study also identified a more stable crystalline structure of Na_3_P and the irreversible consumption of BP during sodiation, leading to capacity loss.^[^ ^18^ ^]^ The third common allotrope of phosphorus, white phosphorus, while a useful synthetic reagent, is highly pyrophoric and toxic and has therefore not been used in electrochemical cells.^[^ ^26^ ^]^

There is extensive research into carbon‐P composites, where carbon structures such as hard carbon (HC), graphite, graphene, or graphene nanotubes are incorporated into P‐based anodes to enhance conductivity and mitigate swelling concerns. Initial studies completed by Marino et al. investigated a ground and heated equal mixture of activated carbon and RP, finding significant improvements in the lifetime of the phosphorus in lithium half‐cells, with ≈900 mAh g^−1^ being seen after 20 cycles.^[^ ^27^ ^]^ Further work using ^31^P and ^7^Li ex situ NMR identified two states of Li_3_P in this material; one which forms reversibly at higher voltages due to confinement in the carbon, and one which is irreversibly formed at lower voltages, and which is isolated and not adsorbed on the carbon surface.^[^ ^28^ ^]^ Promising capacities of >1700 mAh g^−1^ were achieved over 30 cycles using a 7:3 phosphorus‐carbon ratio composite produced using ball milling.^[^ ^16^ ^]^ Further modifications using HCs have provided a capacity of 1200 mAh g^−1^ over 1000 cycles. The porosity of hard carbons strongly depends on the synthetic parameters and precursors employed; thus, P adsorption onto and incorporation into hard carbons can vary drastically.^[^ ^27^ ^]^ Beyond hard carbon, phosphorus has been covalently bonded to graphite, sandwiched between graphene layers, or connected via graphene nanotubes.^[^ ^11^, ^25^, ^29^ ^]^ Studies that combine these allotropes of carbon with either red or black phosphorus show high capacities, due to the conductivity and stabilizing effects of carbon structures.

Beyond composites, the doping of phosphorus into carbon, involving the substitution of carbon atoms in HC, graphene sheets, or a graphite lattice, offers a novel approach for developing new materials with potential applications in energy storage or catalysis.^[^ ^30^, ^31^ ^]^ Most research has been concerned with the incorporation of nitrogen or boron into graphite lattices, as the similar size between the dopant atoms and carbon facilitates effective substitution.^[^ ^31^ ^]^ An initial study on developing phosphorus‐doped carbon (PDC) involved the polymerization of an aromatic compound with phosphorus oxide. However, very low levels of doping were present and phosphorus was primarily incorporated as a secondary phase.^[^ ^32^ ^]^ More recent work incorporating phosphorus atoms into either graphite or hard carbons has predominantly involved the pyrolysis of a carbon source, such as benzene, with a secondary phosphorus source, such as triphenylphosphine, phosphoric acid, or phosphorus trichloride. Studies are often conducted to assess the ability of PDCs to catalyze the oxygen reduction reaction, rather than as lithium‐ or sodium‐intercalation materials.^[^ ^30^, ^33^, ^34^, ^35^ ^]^

Doping of nitrogen or boron into carbon to achieve stoichiometric ratios as high as C_3_E can occur (where E is N or B);^[^ ^36^, ^37^ ^]^ however, an approximate composition of C_5_P has been observed to be the maximum possible incorporation of P into graphite when utilizing benzene and PCl_3_ as pyrolysis precursors.^[^ ^35^ ^]^ The ^31^P NMR results presented in this work were similar to those by Matthews et al. who utilized a bis‐substituted aromatic phosphine as a precursor, showing a mixture of P‐C, P_4_, and phosphate environments, suggesting that the maximum dopant P incorporation may be even lower.^[^ ^38^ ^]^ Matthews reported a first cycle capacity of ≈650 mAh g^−1^ for lithium half‐cells, resulting in the consumption of P_4_ present in their materials during lithiation, contributing to the overall capacity.^[^ ^38^ ^]^ DFT studies have strongly suggested the ability of PDC to intercalate Na^+^ ions, as dopant P atoms can donate electrons to the carbon atoms, enhancing ion‐carbon interactions and thus improving intercalation.^[^ ^39^ ^]^ However, the exact processes by which ion intercalation occurs have not been studied extensively experimentally.

Herein, we present an ex situ solid‐state NMR (SSNMR) study of the lithium and sodium intercalation pathways into phosphorus‐doped carbons. The carbon microspheres formed through single‐step pyrolysis of an organic precursor, containing a number of different electrochemically active phosphorus environments sustain capacities of 400 mAh g^−1^ for lithium and 150 mAh g^−1^ for sodium over 40 cycles. For both systems, the state of the material was examined at various voltages chosen over two cycles to identify the stages of intercalation. ^7^Li, ^23^Na, and ^31^P SSNMR reveal the initial, irreversible consumption of P_4_ followed by a reversible production of Li_3_P or Na_3_P, along with the utilization of P incorporated into the carbon lattice.

## 2 Results and Discussion

### 2.1 Synthesis and Structure of Phosphorus‐Doped Carbons

**Figure 1:**
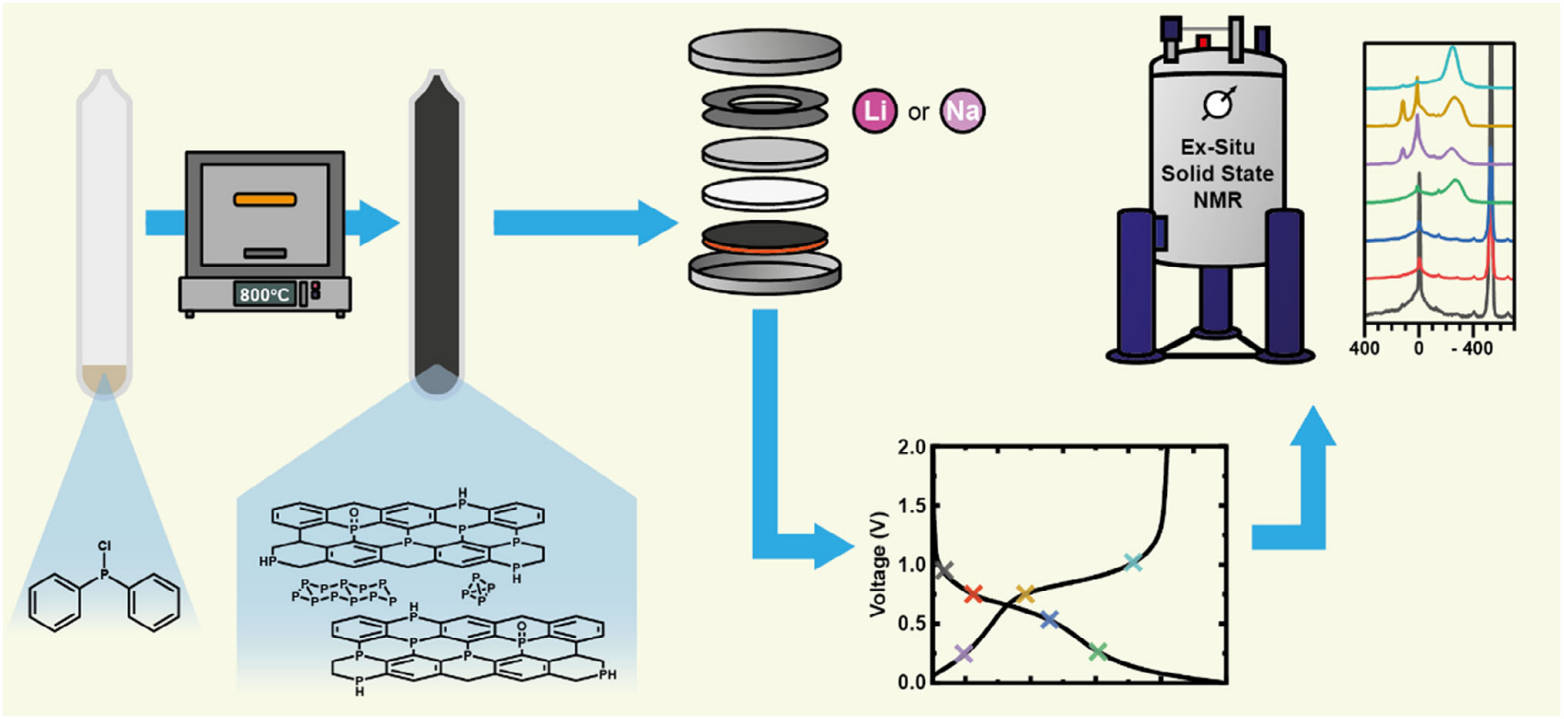
Phosphorus‐doped carbon material was formed through pyrolysis of chlorodiphenylphosphine at 800 °C for 9 h in a quartz ampoule. This material was cycled in half‐cells against lithium or sodium to a specific voltage and characterized using ex situ solid‐state NMR to understand the ion storage mechanics.

**Figure 2:**
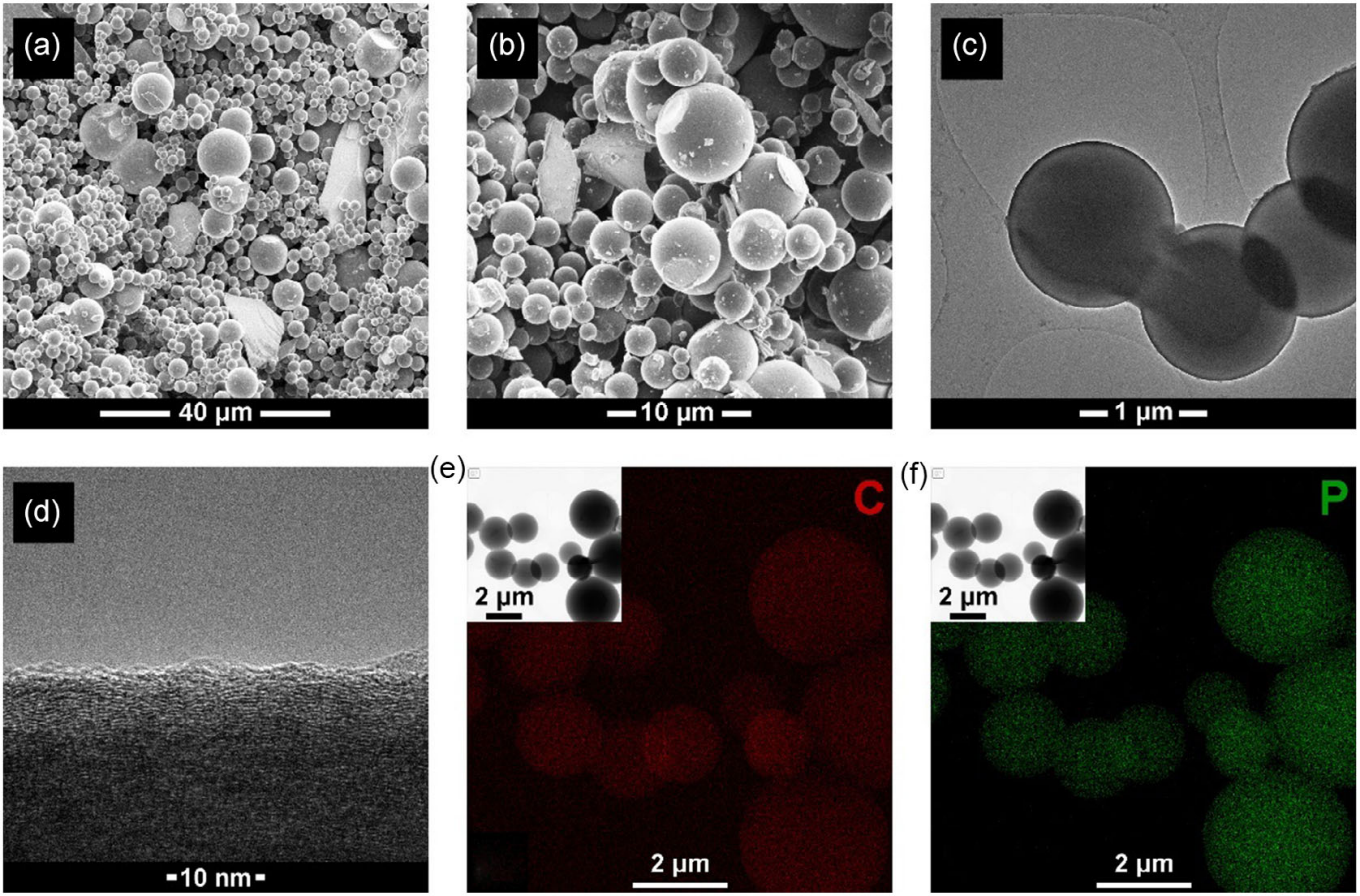
SEM images of a) the bulk material and b) a closer view of the spheres the material is composed of; c) TEM image of the spheres; d) TEM image of the surface of a sphere, demonstrating an amorphous carbon surface layer, covering a turbostratic bulk; EDX analysis showed uniform distribution of both e) carbon and f) phosphorus.

**Figure 3:**
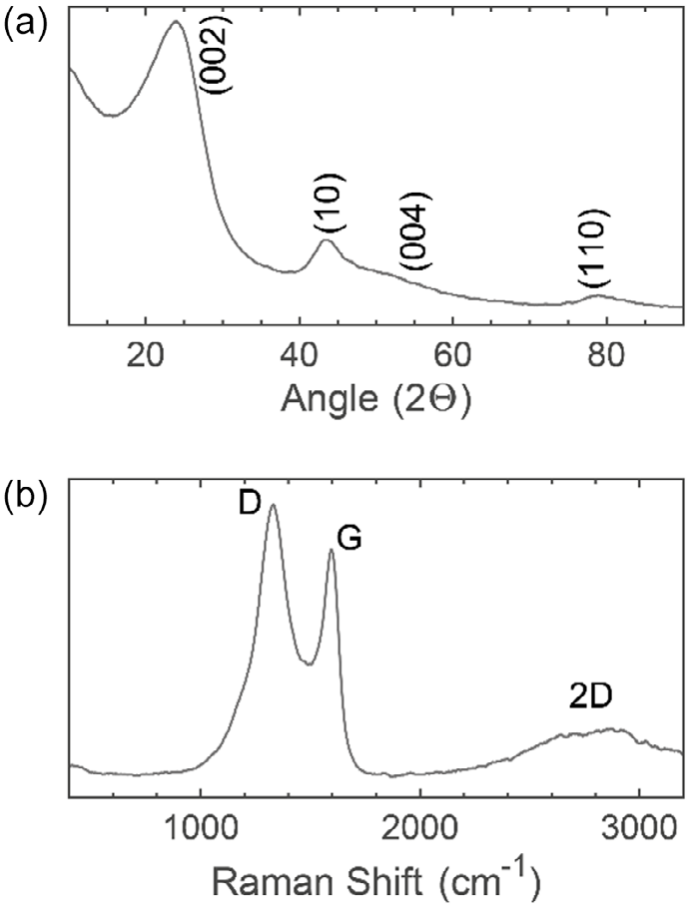
a) XRD pattern and b) Raman spectrum of PDC showing the disordered nature of the material.

Pyrolysis of chlorodiphenylphosphine in a quartz ampoule at 800 °C for 9 h (**Figure** **1**) produced a material primarily consisting of carbonaceous microspheres, 2–10 μm in diameter, together with a smaller fraction of carbonaceous sheets. This can be seen in scanning electron microscopy (SEM) and transmission electron microscopy (TEM) images (**Figure** **2**a–d). At the surface, the spheres appear to have a ≈2 nm layer of amorphous carbon, below this turbostratic layering is observed (Figure 1d). Energy‐dispersive X‐ray spectroscopy (EDS) shows an even distribution of phosphorus across the spheres (Figure 1e,f) and after stoichiometric removal of SiO_2_ an estimated %P of 6.8 at% and a %O of 2.8 at% was determined, corresponding to a % mass of 15.4% and 3.4% respectively. X‐ray diffraction (XRD) (**Figure** **3**a) shows a typical pattern for disordered carbonaceous materials, with a clear (002) peak and (10) region (containing the 100 and 101 peaks). Pawley refinement on XRD data gives an interlayer spacing of 0.369 nm (compared to 0.335 nm for pure graphite^[^ ^40^ ^]^) and a coherence length of 1.1 ± 0.1 nm, indicating a low level of crystallinity and expanded carbon sheets due to the presence of P (as both dopant and elemental phosphorus) and the low temperatures/method used in the synthesis. Raman spectroscopy (Figure 3b) also showed a typical pattern for turbostratic graphitic carbons. Broad D (average position 1331.6 ± 3.9 cm^−1^) and G (average position 1590.8 ± 4.0 cm^−1^) peaks corresponding to the breathing mode of six‐atom rings, and the *E*_2g_ modes, are observed.^[^ ^41^ ^]^ An average *I*(*D*)/*I*(*G*) ratio of 1.22 ± 0.06 was calculated. These results indicate a low level of long‐range ordering throughout the material, which contains turbostratic nanocrystalline graphite domains formed as the precursor decomposes.

**Figure 4:**
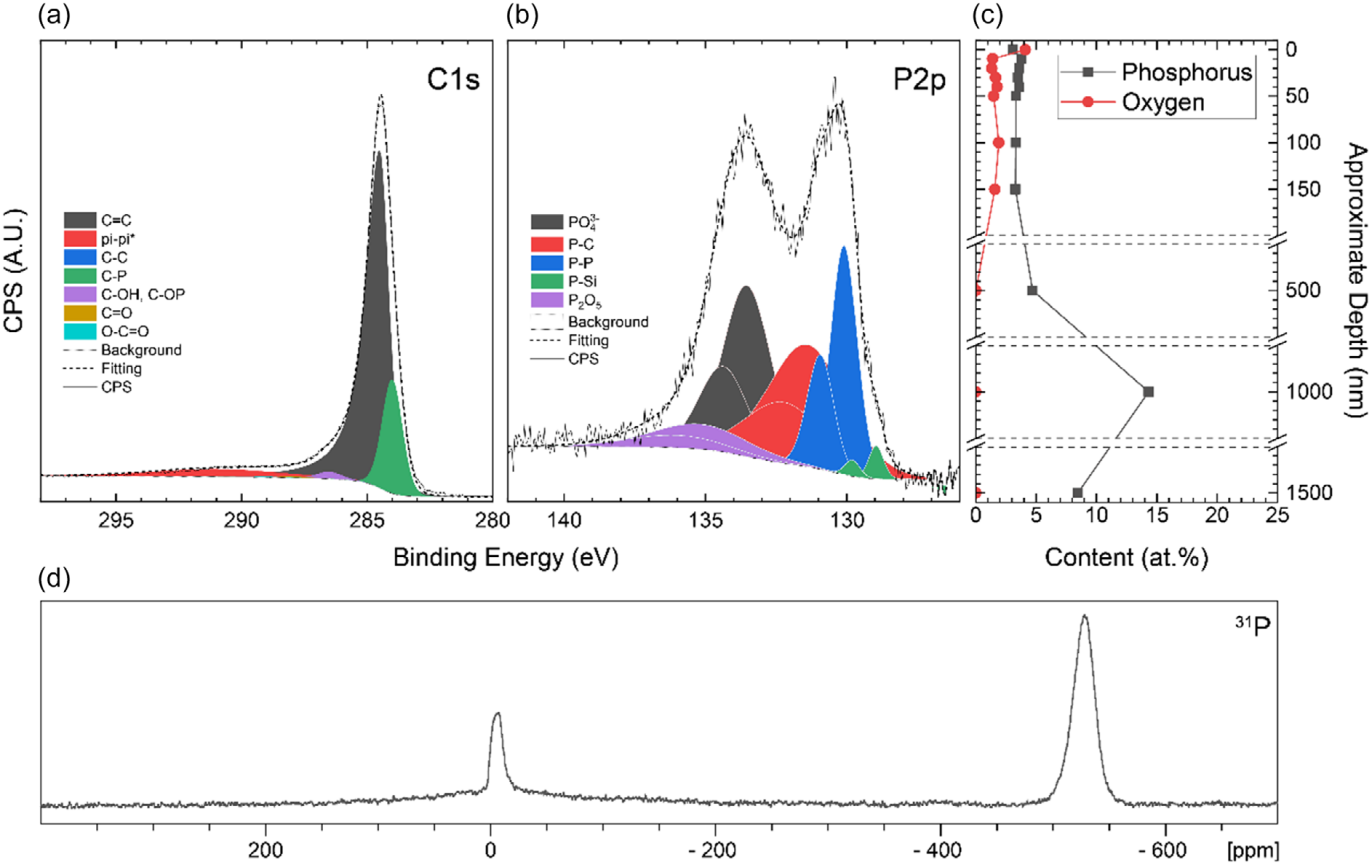
XPS analysis showing high‐resolution spectra of the surface of the material with fitted peaks for the a) C1*s* region and b) P2*p* region. Spin‐orbit coupling was taken into account for the P2*p* region giving two peaks (P2*p* 3/2 and P2*p* 1/2) separated by 0.84 eV for each environment. c) The phosphorus and oxygen atomic % after stoichiometric removal of SiO_2_ calculated from survey spectra. The remaining percentage is carbon. d) ^31^P solid‐state NMR spectrum of pristine PDC (spinning speed, *ν*_rot_ = 25 kHz).

The ^31^P SSNMR spectrum of the pristine material (**Figure** **4**d) shows two distinct environments. The first, at close to 0 ppm, is ascribed to both P covalently bonded to carbon and phosphates.^[^ ^35^, ^42^, ^43^ ^]^ The second environment at −500 ppm is assigned to white phosphorus, present as 45% of the total phosphorus content. Considering the pyrophoric nature of white phosphorus, and the air‐stable property of this material, the elemental phosphorus is assumed to be trapped in closed pores or encapsulated between the carbon layers, protected from the external environment. A broad hump in the ^31^P SSNMR spectrum spanning the region 200 to −200 ppm corresponds to poorly defined phosphorus environments. A ^31^P measurement on a phosphoric acid reference sample allows the calculation of the average mass percentage of phosphorus in PDC as 1.6 ± 0.3%. This value is quite different from the 15.4% estimate from EDS, which may be due to the unreliability of EDS for cases when light elements (such as carbon) contribute significantly to the overall composition of the sample.^[^ ^44^ ^]^

X‐ray photoelectron spectroscopy (XPS) coupled with ion etching allows the measurement of atomic concentration and environments throughout the material (Figure 4a–c). For all datasets, SiO_2_ was stoichiometrically removed, as it is presented as a trace impurity from the quartz reaction vessel. However, some reduced silicon oxide, peaks due to silicon‐phosphorus bonding, and an unidentified peak at 105 eV were also observed as minor components in the high‐resolution P2*p* (Figure S2.1, Supporting Information) and Si2*p* spectra (Figure S2.2, Supporting Information).

Measurements down to a depth of 1.5 μm showed a low quantity of phosphorus present (Figure 4c), with an average atomic % of C: 93.6 ± 2.7 at%, P: 5.0 ± 3.3 at%, and O: 1.4 ± 1.1 at%, corresponding to a C:P ratio of 18.7:1. Full survey spectra can be found in Figure S2.3, Supporting Information.

The oxygen that is observed in the XPS comes from the quartz tube used in the synthesis (SiO_2_), oxygen species (including water) that are physisorbed to the carbon surface, and also from reaction with exposed phosphorus groups that may functionalize the surface (see below). High‐resolution XPS of the C1s region (Figure 4a) indicates that the majority of carbon detected is *sp* ^2^ bonded C=C. A C—P bonding component and a very minor quantity of various carbon–oxygen bonding components are also seen, along with an *sp* ^3^ C—C bonding component. Environments were generally similar in relative quantity at each depth studied (Figure S2.1, Supporting Information).

The high‐resolution P2*p* region shows a variety of phosphorus environments present (Figure 4b). While a moderate amount of C—P bonding was detected, phosphate environments were also present. These are possibly formed through oxidation of surface phosphorus during washing or through reaction with the quartz tube at high temperatures during annealing. It can be noted, however, that the presence of these environments below the surface suggests they are formed during synthesis. It is also worth noting that there is the possibility of a wider variety of minor environments which may overlap and contribute to the overall line shape (such as Ph_*x*_PO_*y*_, *x* + *y* = 5).

To avoid over‐fitting, phosphorus environments initially clearly identified in the ^31^P solid‐state NMR (SSNMR) spectra of the pristine material (Figure 4d) were fitted as components in the P2*p* region. A further Si—P bonded component (observed in the Si2*p* spectra) was then added. Spin‐orbit coupling was accounted for, fitting a second peak for each environment at +0.84 eV with half the areas and equal full‐width half‐maxima.

### 2.2 Lithium‐Ion Half‐Cell Systems

**Figure 5:**
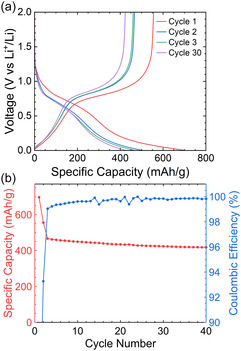
Performance of phosphorus‐doped carbon in lithium‐ion half cells. a) The first, second, third and thirtieth cycle of galvanostatic charge‐discharge (GCD) curves at C/20 within a potential range of 0.005–2 V versus Li^+^/Li using 1 m LiPF_6_ in EC/DMC (50/50 v/v); b) cycling performance at a rate of C/20 (92 mA).

The material gives excellent performance as an electrode in lithium half‐cells, sustaining a specific capacity of 416 mAh g^−1^ after 40 cycles and a coulombic efficiency of >99.8%. In the voltage profile (**Figure** **5**a), two plateaus are identified, at ≈0.7 and 0.2 V versus Li^+^/Li.

A comparison of the experimental cell‐specific capacity with calculated specific capacity using the mass % values (determined by EDS and SSNMR) and the atomic % values (determined by XPS) are shown in **Table** **1**. Full calculations of these values can be found in Table S1, Supporting Information. The calculated theoretical specific capacity values in Table 1 compared to the experimental value of 416 mAh g^−1^ indicate the % mass of phosphorus determined through SSNMR is likely to be the closest to the real value. The full calculations also reveal that for the atomic ratio determined through SSNMR, the carbon atoms make a greater contribution to the experimentally observed capacity than the phosphorus atoms.

**Table 1:** Summary comparison of mass and atomic ratios, and theoretical specific capacity determined through characterization technique results. Full calculations can be found in Table ST1, Supporting Information.

	EDS 2	SSNMR	XPS
C:P mass ratio	5.5:1	61.5:1	7.3:1
C:P atomic ratio	14.2:1	158:1	18.7:1
Theoretical specific capacity [mAh g^−1^]	710.3	407.2	637.7

**Figure 6:**
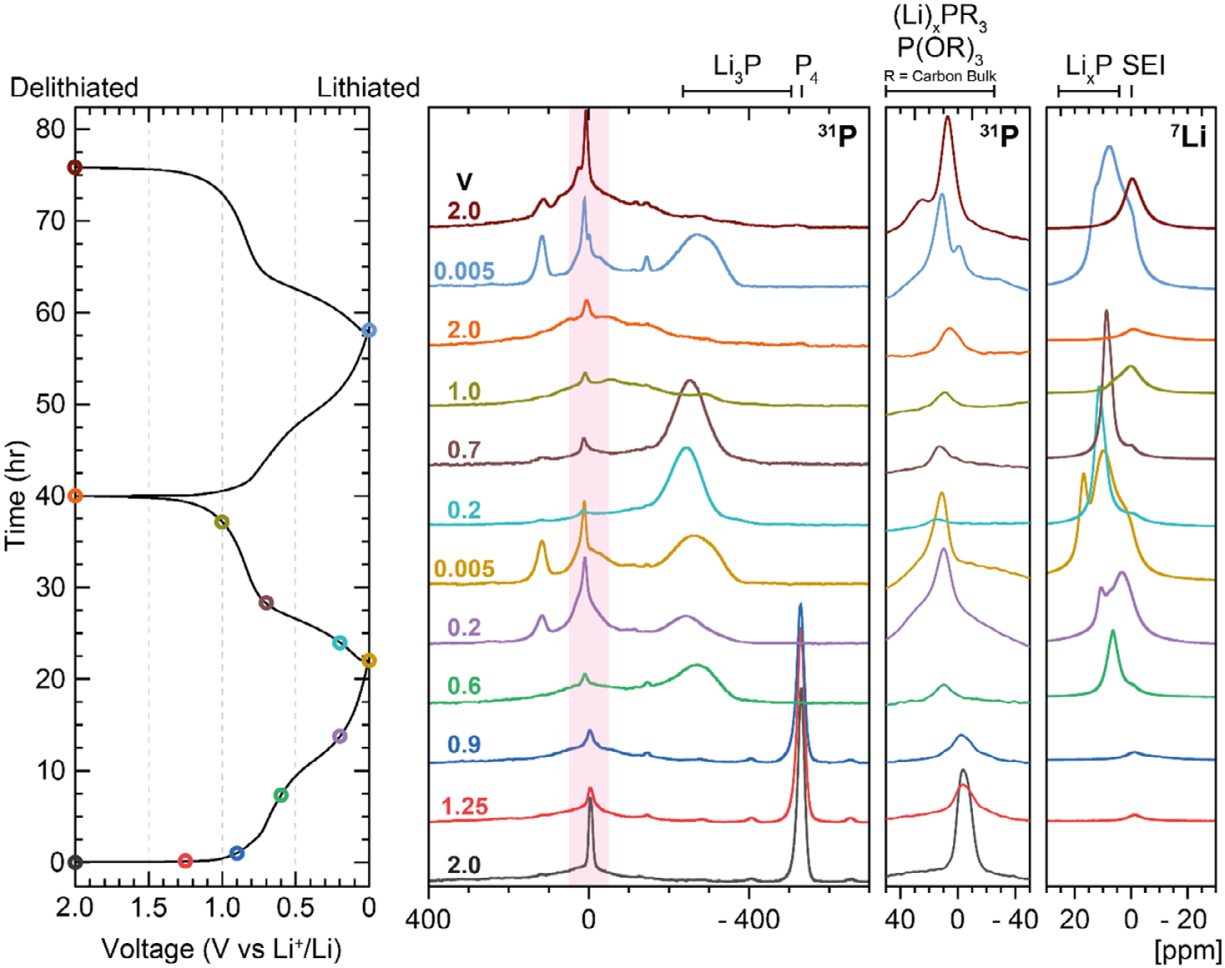
Voltage profile and corresponding ex situ ^31^P and ^7^Li SSNMR spectra for PDC in lithium‐ion half cells stopped at specific voltages during cycling. Expansions of the region of the ^31^P spectra (shaded in pink in the left‐hand side ^31^P spectra) containing the phosphate environments are shown to the right of the full ^31^P spectra. (*ν*_rot_ = 25 kHz). Cells were cycled at C/20 within a potential range of 0.005–2 V versus Li^+^/Li using 1 m LiPF_6_ in EC/DMC (50/50 v/v).

**Figure 7:**
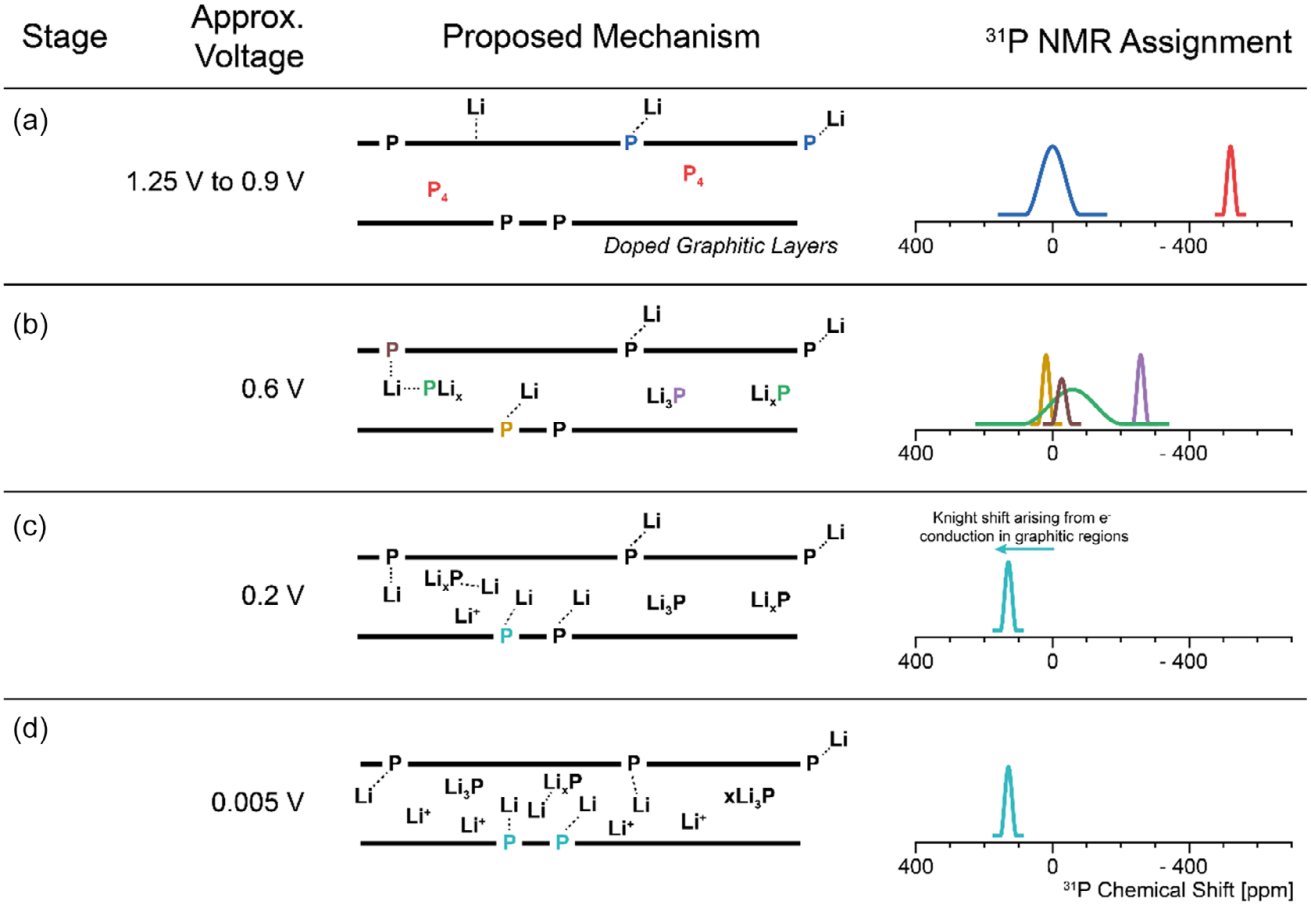
Proposed mechanism for the lithiation of PDC (center, carbon sheets are shown as bold lines). The approximate voltage for each stage a–d) of the mechanism is shown on the left. On the right, ^31^P shift ranges of new species formed at each stage are shown, color coded according to the related environment shown to the left. These proposed shifts and assignments are based on the ^31^P NMR spectra in Figure 6. Most of the insertion processes between (c) 0.2 and (d) 0.005 V involve lithiation of more carbon‐rich regions; hence, no new ^31^P resonances are observed.

Ex situ ^31^P and ^7^Li SSNMR (**Figure** **6**) show how the phosphorus species evolve at different voltages. The lithiation processes occurring are likely to be highly complex, and one possible mechanism for the observed electrochemical behavior is outlined in **Figure** **7**, which we propose based on the ex situ NMR studies. Initially (Figure 7, panel a and Figure 6, 1.25–0.9 V versus Li^+^/Li), a very minor quantity of lithium is inserted and interacts weakly with phosphorus covalently bonded with carbon, likely at the edges of the graphitic structures. By analogy with hard carbons, some capacity seen at higher voltages can be assigned to intercalation into nearby defects in the carbon sheets. Here, some of these carbon defects are nearby or caused by P, based on the broadening and reduction of the intensity of the ^31^P peak at close to 0 ppm.

White phosphorus is consumed from 0.9 to 0.6 V versus Li^+^/Li (Figure 7b) producing a variety of Li_*x*_P compounds, primarily local environments close to those found in Li_3_P, as seen by the emergence of a peak centered at −270 ppm.^[^ ^12^, ^28^, ^38^ ^]^ In the ^7^Li spectra (Figure 6, right), the appearance of encapsulated Li_*x*_P species is observed at 6.5 ppm, in rough agreement with literature values.^[^ ^28^, ^45^ ^]^ Since the P_4_ is encapsulated within the carbon structure, Li_3_P must be present in small clusters containing at least four P atoms within the carbon. The asymmetry of the ^31^P peak associated with Li_3_P (δ(^31^P) = −270 ppm, Figure 6, center) can be attributed to varying degrees of interaction between the Li_3_P clusters and the carbon surfaces, variations in the size of the Li_3_P clusters, and also Li_*x*_P environments where *x* < 3.

The doped‐phosphorus environments interact with Li^+^ present between the graphitic sheets (δ(^31^P) = ≈0 ppm). Furthermore, we speculate that Li‐bridged structures involving Li_3_P (δ(^31^P) ≈ 0–50 ppm, Figure 6, center) may form as schematically illustrated in Figure 7b,c.^[^ ^46^ ^]^

At 0.2 V versus Li^+^/Li, the ^7^Li peaks for the Li_*x*_P and lithiated P‐doped carbon species split and two peaks centered at 3.0 and 10.9 ppm appear (with a weak shoulder at 6.5 ppm). We assign these additional peaks to lithiated doped‐graphite and environments similar to those found in lithiated undoped graphite (e.g., dilute stage 2, LiC_18_), respectively.^[^ ^47^, ^48^, ^49^ ^]^ It should be noted that the shifts typically seen for high‐lithium content graphite intercalation compounds (LiC_12_ and LiC_6_, approx. 42 – 45 ppm) are never observed here at the expected lower voltages.^[^ ^48^, ^50^ ^]^ These new ^7^Li resonances are associated with the appearance of a new peak in the ^31^P spectrum at ≈120 ppm that does not correspond to literature‐known species. In previous work, LiP_7_ was the only Li_*x*_P species predicted to have a positive ^31^P shift; however, this species would be formed at a higher voltage versus Li^+^/Li than here. Higher lithium content Li_*x*_P compounds were predicted only in the range δ(^31^P) ≈ 0 to −270 ppm.^[^ ^12^ ^]^ We propose that this phosphorus environment is near carbon atoms and surrounded by lithium ions inserted into the bulk graphitic structure. We tentatively assign the positive shift to a Knight shift arising from conduction electrons in the more graphitic regions, by analogy with shifts seen for ^7^Li in graphite intercalation compounds.

At a voltage of 0.005 V versus Li^+^/Li, the ^7^Li SSNMR spectra clearly show that further lithium insertion occurs. A ^7^Li peak at ≈17 ppm develops, assigned to lithium intercalation into disordered graphitic‐like parts of the material and possibly Li^+^ nearby P within the carbon sheets; additionally, the other peaks relating to Li–P interactions grow in intensity.^[^ ^47^, ^49^, ^51^ ^]^ The weak growth of the peak at ≈120 ppm (Figure 7d) and only minor changes to the other ^31^P peaks suggest that most of the reduction involves the graphitic regions. At 0.005 V versus Li^+^/Li, it is possible that “pooling” of lithium to form small lithium metal clusters in the hard carbon (HC) regions could occur; however, no ^7^Li resonances are seen at frequencies of between 60 and 245 ppm, where the Li pooled Knight‐shifted peaks are generally observed, confirming that our materials do not contain internal porosity (that can be accessed by Li^+^ ions).

Upon initial delithiation to 0.2 V versus Li^+^/Li, the peaks in the ^7^Li spectrum at 17.1 and 3.4 ppm disappear, along with the ^31^P resonances at positive frequencies, consistent with the removal of Li^+^ ions in both P‐doped carbon and the more graphitic carbon‐like environments. In the ^31^P spectrum, only the Li_3_P‐like environments remain (at −270 ppm), further suggesting the removal of lithium from phosphorus‐doped carbon environments. At this voltage, the only major peak in the ^7^Li spectra is at ≈11.5 ppm, while previously the ^7^Li resonance assigned to Li_3_P was at 6.5 ppm (at a voltage of 0.6 V versus Li^+^/Li during lithiation). Upon delithiation to 0.7 V versus Li^+^/Li, this ^7^Li peak shifts to a lower frequency (8.7 ppm) approaching the shift seen during lithiation. The difference between lithiation and delithiation is reminiscent of the hysteresis seen in many conversion reactions and suggests that the phases (or small clusters) seen on delithiation may be written as Li_3‐x_P while those on lithiation may be richer in lithium. The solitary peak in the ^7^Li spectrum at 8.7 ppm disappears between 0.7 and 1 V versus Li^+^/Li. This disappearance correlates with the disappearance of the Li_3_P peak in the ^31^P spectra confirming that the peak at 8.7 ppm at 0.7 V versus Li^+^/Li can indeed be assigned to Li_3_P. The changes in the electronic structure of the carbon sheets may also affect the ^31^P resonances as seen for example by the small changes seen in the “Li_3_P” resonance between 0.005 and 0.2 V; this shift also suggests that the Li_3_P clusters are contained within the carbon sheets.

As the delithiation of Li_3_P occurs, between 0.7 and 1.0 V versus Li^+^/Li, a very broad resonance centered at δ(^31^P) 0 ppm is observed to form. Given that red phosphorus gives rise to a peak at ≈δ(^31^P) 50 ppm,^[^ ^52^ ^]^ we suggest that amorphous P is reformed with a local structure that is closer to that of red phosphorus, rather than the white phosphorus that was present in the pristine material. Marino et al. identified two forms of Li_3_P when red P was confined in porous carbon, “adsorbed” (δ(^7^Li) = ≈6 ppm) and “not adsorbed” (δ(^7^Li) = ≈4.5 ppm). Their study identified that the Li associated with the latter environment did not cycle reversibly. The apparent total reversible lithiation of elemental phosphorus present, along with the lack of a peak at δ(^7^Li) = ≈4.5 ppm at 0.005 V versus Li^+^/Li, suggests that all of the Li_3_P present in our material is adsorbed internally on carbon structures and thus electrically connected. Total delithiation to 2.0 V resulted in little change in the ^31^P spectrum and gave a ^7^Li spectrum only containing signals that can be assigned to SEI resonances.

The second lithiation to 0.005 V versus Li^+^/Li gave rise to the previously observed ^7^Li and ^31^P peaks in albeit slightly different intensities: a stronger peak in the ^31^P spectra at 0 ppm with a shoulder (assigned to lithiated dopant phosphorus as in the previous cycle), and a residual electrolyte peak at ≈−145 ppm were seen.^[^ ^53^ ^]^ Upon a second delithiation to 2.0 V, amorphous red phosphorus‐like environments are reformed, resulting in a broad hump in the ^31^P spectra spanning 200 to −200 ppm.^[^ ^52^ ^]^ However, the ^31^P peak at ≈120 ppm partially remains, and the peak at 10 ppm appears more intense. This suggests that lithium that is part of the lithiated doped‐phosphorus environments becomes trapped, contributing in part to cell capacity degradation. Degradation may also come from decomposition of the electrolyte reacting with active phosphorus on the surfaces of the material. Furthermore, at a second delithiation to 2.0 V, a ^7^Li peak at 0 ppm is observed also indicating residual electrolyte and SEI are present; the peak is broad enough to partially encompass the region where residual lithiated doped‐phosphorus may appear.

We note that in order to check for any relaxation/degradation that may occur within lithiated PDC between cycling and examination using SSNMR, a sample was left packed in a nominally airtight rotor that was left in the air for 14 days. A comparison of this with a sample examined using SSNMR immediately after cycling is shown in Figure S4, Supporting Information. The spectra indicate that the ^31^P environments in the samples do not undergo any degradation or relaxation between ex situ NMR analysis performed immediately after cycling and after 14 days. Finally, we suggest that further density functional theory (DFT) calculations and heteronuclear NMR experiments may help provide additional insight into the nature of the wide range of local environments seen in these systems, but the assignments presented above have attempted to reconcile the results from many of the previous studies performed on carbon‐ and phosphorus‐only systems and different P/C composites.

### 2.3 Sodium‐Ion Half‐Cell Systems

**Figure 8:**
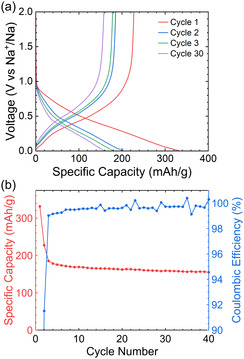
Performance of phosphorus‐doped carbon in sodium‐ion half cells. a) The first, second, third and thirtieth cycle of GCD curves at C/20 within a potential range of 0.005–2 V versus Na^+^/Na using 1 m NaPF_6_ in EC/DMC (50/50 v/v); b) cycling performance at a rate of C/20 (118 mA).

The material performed moderately well as the anode in sodium‐ion half‐cells, maintaining a capacity of 160 mAh g^−1^ after 40 cycles (**Figure** **8**). The voltage profile was poorly defined compared to the lithium system; however, two distinct stages, above and below 0.5 V versus Na^+^/Na, may still be distinguished.

Capacity from the sloping region is assigned to both the intercalation of sodium into disordered and defected graphene‐like layers (including heteroatom‐doped regions), and from the reaction of sodium with phosphorus present within the material. No flat, plateau‐like region, just above 0 V versus Na^+^/Na, typical of sodiation (and lithiation) of hard carbons is seen.^[^ ^54^, ^55^, ^56^ ^]^ This is likely a result of the low annealing temperature and suggests that the material is highly disordered, not containing voids that are suitable for the “pooling” of metallic sodium.^[^ ^6^, ^56^, ^57^ ^]^ This furthermore suggests that any Na in any voids must interact with the carbon sufficiently strongly so that some partial charge transfer occurs. This proposal is now explored via NMR spectroscopy.

**Figure 9:**
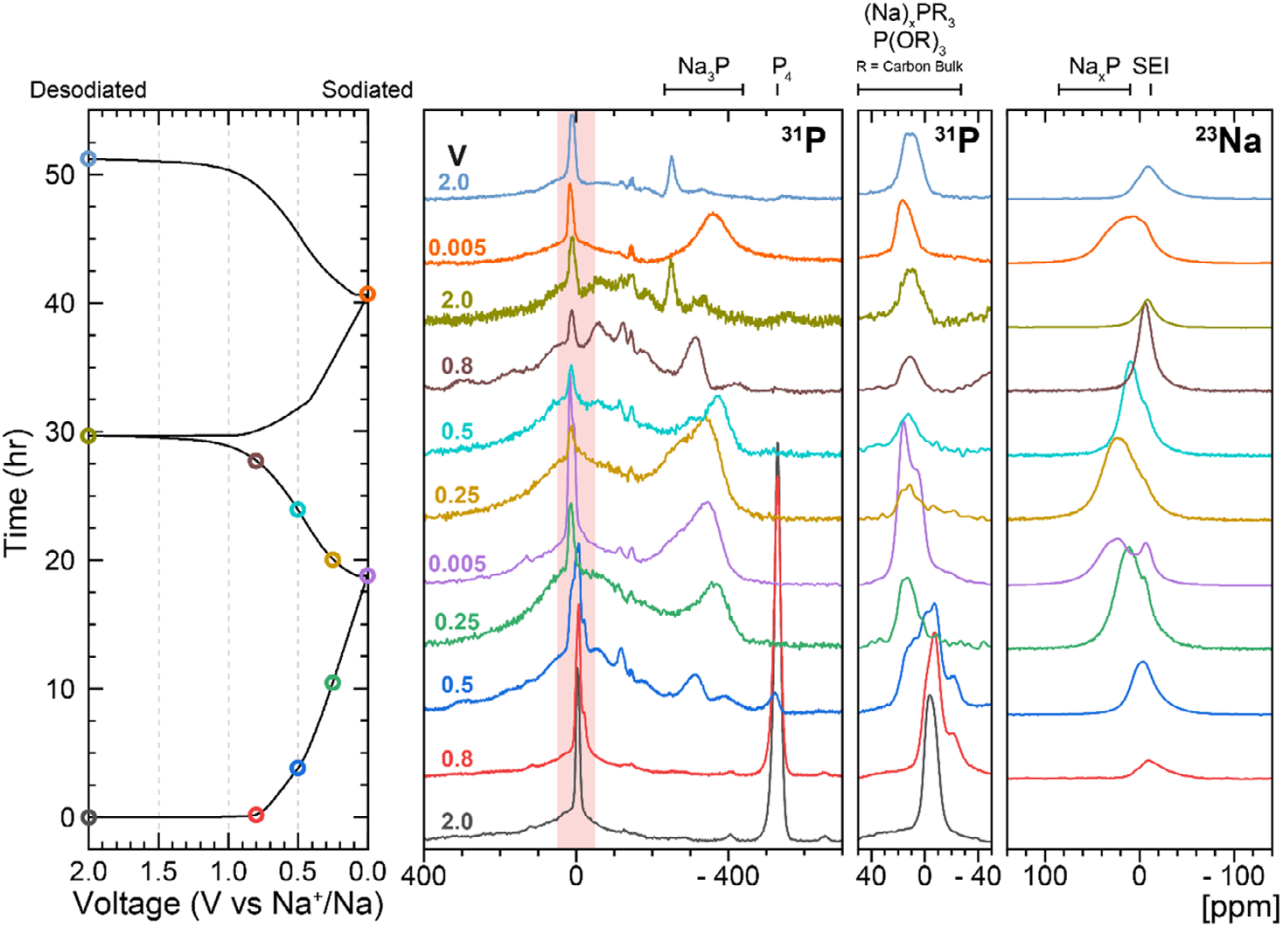
Voltage profile and corresponding ex situ ^31^P and ^23^Na solid‐state NMR spectra for PDC in sodium‐ion half cells stopped at specific voltages during cycling. (*ν*_rot_ = 25 kHz). Cells were cycled at C/20 within a potential range of 0.005–2 V versus Na^+^/Na using 1 m NaPF_6_ in EC/DMC (50/50 v/v).

Ex situ ^31^P and ^23^Na SSNMR measurements (**Figure** **9**) revealed that the processes occurring during cycling are qualitatively similar to those in the lithium system. Initially, between 0.8 and 0.5 V versus Na^+^/Na, the P_4_ is consumed, forming a variety of amorphous Na_*x*_P compounds. These appear as a broad, overlapping series of resonances spanning 200 to −200 ppm (centered on ≈−30 ppm) along with broad lower frequency resonances at ≈−310 and −400 ppm in the ^31^P spectrum at 0.5 V versus Na^+^/Na. Further analysis is complicated by overlapping spinning side bands. The species present are most likely to be a series of amorphous Na–P phases with compositions close to Na_3_P_11_, Na_3_P_7_, and NaP, as the ^31^P NMR chemical shifts and formation voltages are in reasonable agreement with the range of shifts predicted (and seen experimentally) in the literature.^[^ ^12^, ^18^ ^]^ The ^23^Na NMR spectra also show the development of Na_*x*_P species between 0 and 45 ppm as the voltage decreases, in agreement with the literature.^[^ ^18^ ^]^ From 0.5 to 0.25 V versus Na/Na^+^, the higher frequency region in the ^31^P spectra associated with amorphous Na–P_*x*_ phases continued to grow, while simultaneously an intense, asymmetric peak grows at ≈−340 ppm. From 0.25 to 0.005 V, the peaks at the low frequencies grow, while the intensities of the peaks in the broad region centered on ≈−30 ppm shrink. These observations are ascribed to the formation of increasingly sodiated Na–P phases, which are then converted to amorphous/nanoparticulate Na_3_P. Crystalline Na_3_P resonates at ≈−207 ppm^[^ ^12^, ^18^ ^]^ and the differences in shifts observed here are attributed to encapsulation of Na_3_P in carbon, which prevents the crystallization of Na_3_P. It is likely a range of different local environments are formed, including residual P‐P clusters.

The peak tentatively assigned to P doped in the carbon sheets (δ(^31^P) = 0 ppm) broadens and shifts to higher frequency as the sodiation occurs. However, unlike the lithium system, a ^31^P peak at positive frequencies (which we assigned to P within the carbon sheets, shifted to higher frequency due to a Knight shift) is not observed. Sodium is not readily inserted in graphite and we suggest that it similarly does not insert into the more ordered graphitic regions of our material, even if they are partially disordered by the introduction of P dopants. This explanation is consistent with the lower capacity of the Na‐system, particularly in this voltage range. No ^23^Na resonances are observed in the region between 700 and 960 ppm, typically seen for “pooled” Na metallic clusters in hard carbons, confirming that all the intercalated Na must interact at least to some degree with the carbon or P in the system.^[^ ^55^ ^]^

Upon desodiation, essentially no changes in the ^31^P spectra in the region assigned to amorphous Na_3_P‐like species are observed between 0.005 and 0.25 V versus Na^+^/Na. A reduction in the intensity of the P dopant peak at δ(^31^P) 0 ppm is observed, suggesting that there is some carbonaceous bulk material that is sufficiently disordered (and reminiscent of a HC) such that it is capable of a small uptake of sodium. The capacity in this voltage range is, however, lower than seen in the Li region, consistent with the above proposal that Na is not inserted into the more ordered (P‐doped) graphitic regions. At 0.5 and then 0.8 V versus Na^+^/Na, the Na_3_P peak begins to shrink in intensity as a number of peaks appear below 0 ppm. This corresponds to the formation of the aforementioned lower sodium content Na_*x*_P compounds.^[^ ^12^ ^]^ Upon total desodiation at 2.0 V versus Na^+^/Na, a complex spectrum remains, with two more resolved peaks at 10 ppm (dopant phosphorus) and a peak at −240 ppm that remains unidentified. The ^31^P resonance at −240 ppm has a similar chemical shift to PH_3_;^[^ ^58^ ^]^ however, a ^1^H NMR spectrum of this sample did not show the presence of PH_3_ (Figure S3, Supporting Information). The white phosphorus, as in the lithium system, is not reformed, and instead, a very broad feature close to 0 ppm is seen which we assign to amorphous red P. In the ^23^Na NMR spectra at 2.0 V versus Na^+^/Na, a single peak centered at ≈−8 ppm is present, assigned to compounds resulting from SEI formation.

The ^31^P spectrum following the second sodiation to 0.005 V versus Na^+^/Na (Figure 9, orange spectrum) presents a clear, more symmetric amorphous or partially crystalline Na_3_P peak (δ(^31^P) = −270 ppm), a (sodiated) dopant P peak (δ(^31^P) = 10 ppm), and a minor peak corresponding to residual NaPF_6_ electrolyte (δ(^31^P) = ≈−145 ppm). A second desodiation to 2.0 V versus Na^+^/Na results in a cleaner spectrum than that from the first cycle, with the same set of well‐resolved peaks present. Postcycling analysis of these cells revealed a substantial SEI (Figure S1.2, Supporting Information) and discolored (orange) electrolyte, suggesting capacity loss may come from the formation of the SEI and related degradation of the electrolyte. Further studies are required to identify the exact mechanisms of electrolyte degradation in this case.

## 3 Conclusion

In this work, we have for the first time determined the changes that occur in phosphorus‐doped and encapsulated carbons during electrochemical cycling in both lithium and sodium cells. Although a number of other studies have identified the doped‐P and white P environments present in pyrolytic P‐doped carbons, to our knowledge, none have looked in detail at electrochemical changes and identified species present during cycling. Here, we use a facile synthetic technique which allows the production of large quantities of phosphorus‐doped and encapsulated graphite spheres that show effective cycling capacity and reversibility. We reveal the consumption of the encapsulated P_4_ to form species resembling Li_3_P/Na_3_P between 0.9 and 0.6 V against Li^+^/Li, and 0.8 and 0.5 V against Na^+^/Na, but contained within the carbonaceous structure. We identify that the P‐doped carbon environments and the host carbon structure play an important role in ion storage at low voltages (<0.25 V). Furthermore, we reveal that P_4_, known to be encapsulated in the as‐synthesized material, does not reform upon charging. Instead, an encapsulated amorphous/poorly crystalline phase similar in structure to red phosphorus is produced, which in subsequent cycles reversibly undergoes the transformation P ↔ Li_3_P. Furthermore, we show that the carbon spheres produced by this method, in contrast to hard carbons, do not contain voids suitable for the formation of small Li/Na metallic clusters. On the basis of these results, by making alterations to the synthetic protocol, we are working on optimizing the electrochemical performance of the material with regard to capacity and lifetime.

## 4 Experimental Section

4.1

4.1.1

##### Synthesis of PDC

The fabrication of PDC described here involves the synthesis of hazardous and pyrophoric (white phosphorus) materials inside pressurized, fragile quartz ampoules. These ampoules are liable to detonate during the heating or extraction processes. A high level of caution should be taken not to over‐fill the ampoules and when obtaining the material.

A quartz ampoule (*L*: 100 mm, *Ø*: 25 mm) was charged with chlorodiphenylphosphine (200 mg) (Sigma–Aldrich) under inert atmosphere (N_2_, O_2_ < 0.5 ppm, H_2_O < 0.5 ppm). The ampoule was sealed with a hydrogen/oxygen torch under dynamic vacuum (10^−3^ bar). The ampoules were heated to 800 °C at 10 °C min^−1^ and held at this temperature for 9 h using a Carbolite ELF 11/14B furnace. The material was extracted by scoring around the ampoule using a diamond glass cutter and snapping the ampoules in an inert atmosphere (N_2_, O_2_ < 0.5 ppm, H_2_O < 0.5 ppm). Due to the high pressure built up inside the ampoules, they were wrapped in duct tape and placed inside appropriately sized, thick, braided tubing before snapping. A black needle‐like material was subsequently obtained. This was initially washed in inert atmosphere using dried toluene to remove any exposed P_4_ present. The material was then further washed with water and acetone in air and allowed to dry for 12 h under ambient conditions. The dry material was ground in a pestle and mortar to an homogenous powder. Typically, ≈100 mg of material was obtained.

##### Analytical Techniques

Scanning electron microscope (SEM) images were collected using a Magellan 400 at 2 kV for pristine samples, and a TESCAN MIRA 3 FEG‐SEM at 5 kV for post‐cycling analysis and EDS analysis, using an Oxford Instruments X‐maxN 80 probe. For postcycling SEM/EDS analysis, cells were disassembled in an inert atmosphere (Ar, O_2_ < 0.1 ppm, H_2_O < 0.1 ppm). The electrodes were washed with dimethyl carbonate solvent (anhydrous, >99%, Sigma–Aldrich) to rinse off electrolyte residue and dried in the glovebox. Whole electrodes were used for SEM/EDS analysis, which were taken out of air‐sensitive conditions for transfer from the glovebox.

TEM images were collected using a Thermo Scientific (FEI) Talos F200X G2 TEM.

Raman spectroscopy mapping measurements were performed using a Renishaw InVia with a 2400 L mm^−1^ grating and 512 nm laser. Peak fitting was performed using OriginLab Origin data analysis software using a Gaussian fit. Powder X‐ray diffraction data were collected on a Malvern Panalytical X’Pert Pro instrument, equipped with an X'celerator detector using nonmonochromated CuKα radiation (*λ* = 1.5418 Å). The sample was placed on a glass sample holder and measured in reflection geometry with sample spinning. The data were collected at room temperature over a 2*θ* range of 5–90°, with an effective step size of 0.167° and a total collection time of 20 min. Pawley refinements were executed using TOPAS V6.2.

XPS analysis was performed using a Thermo NEXSA XPS fitted with a monochromated Al kα X‐ray source (1486.7 eV), a spherical sector analyzer, 3 multichannel resistive plate, and 128 channel delay line detectors. All data were recorded at 19.2 W and an X‐ray beam size of 200 × 100 μm. Survey scans were recorded at a pass energy of 160 eV, and high‐resolution scans recorded at a pass energy of 20 eV. Electronic charge neutralization was achieved using a dual‐beam low‐energy electron/ion source (Thermo Scientific FG‐03). Ion gun current = 150 μA. Ion gun voltage = 45 V. All sample data were recorded at a pressure below 10^−8^ Torr and a room temperature of 294 K. Samples were etched using a Thermo MAGCIS ion gun operating in monatomic mode at 4000 eV and a raster area of 2 mm^2^. Data were analyzed using CasaXPS v2.3.24PR1.0. Peaks were fit with a Shirley background prior to component analysis. Lineshapes of GL(30) were used to fit components.

Solid‐state NMR was performed on a Bruker Avance IIIHD spectrometer equipped with an 11.7 T magnet (ν_0_(^1^H) = 500 MHz, ν_0_(^7^Li) = 194.4 MHz, ν_0_(^23^Na) = 132.3 MHz, ν_0_(^31^P) = 202.5 MHz). A 2.5 mm double channel MAS probe was used with a spinning speed of 25 kHz using a rotor‐synchronized Hahn‐echo pulse sequence. ^1^H, ^31^P, ^7^Li, and ^23^Na spin‐lattice relaxation time constants (*T*_1_) were measured using a saturation recovery experiment and recycle delays were set to 5 times the longest *T*_1_. Chemical shifts were references using adamantane (δ_iso_(^1^H) = 1.85 ppm), lithium chloride (δ_iso_(^7^Li) = 0 ppm), sodium chloride (δ_iso_(^23^Na) = 7.2 ppm), and ammonium dihydrogen phosphate (δ_iso_(^31^P) = 0.8 ppm).

For ex situ NMR studies, cells were disassembled in an inert atmosphere (Ar, O_2_ < 0.1 ppm, H_2_O < 0.1 ppm). The electrodes were washed with dimethyl carbonate solvent (anhydrous, >99%, Sigma–Aldrich) to rinse off electrolyte residue and dried in the glovebox. The material was gently scraped off the current collector and packed into air‐tight rotors. NMR experiments were performed as soon as possible after cells were removed from cycling. This was typically within 24 h. The scaling factor applied to the spectra in Figure 6 and 9 was calculated by normalizing the sample mass and number of scans, such that the peak integral and size in all spectra for each figure are comparable.

##### Cell Fabrication and Electrochemical Cycling

PDC was mixed with super‐P Carbon Black (2%) and polyvinylidene fluoride (PVDF) (6%) in N‐methyl‐2‐pyrrolidone (NMP) and homogenized in an Intertronics ThinkyMixer at 2000 rpm for 10 min. The resulting slurry was spread over copper foil at a thickness of 150 μm, dried in air at 60 °C, and then under vacuum at 100 °C. Electrodes (Ø = 12.7 mm) were cut from the material and dried in air at 60 °C for 24 h, and then under vacuum (10^−3^ bar) at 100 °C. Cells were assembled under an inert atmosphere (Ar, O_2_ < 0.1 ppm, H_2_O < 0.1 ppm).

The electrodes were tested in CR2032 coin cells in lithium or sodium half‐cell configurations. The electrolyte was either LiPF_6_ or NaPF_6_, for lithium or sodium cells respectively, in a 1:1 volume ratio of ethylene carbonate (EC):dimethyl carbonate (DMC) at 1 m concentration. The counter electrode was either pure lithium or sodium metal as appropriate. The separator was Whatman 1 glass fiber. Cells were rested for 24 h to ensure that internal chemical processes had stabilized, to achieve consistent electrolyte soaking, and to eliminate any electrolyte concentration gradient in the cell. Cells were then cycled at C/20, calculated using a theoretical specific capacity of graphite of 372 mAh g^−1^ for lithium cells and 300 mAh g^−1^ as an estimation for sodium cells.

## Conflict of Interest

The authors declare no conflict of interest.

## Supporting information

Supplementary Material

## Data Availability

The data that support the findings of this study are available from the corresponding author upon reasonable request.
