# Supplementary material for: Solid‐State Nuclear Magnetic Resonance Investigations of the Lithium‐ and Sodium‐Storage Mechanisms of Pyrolytic Phosphorus‐Carbon Composites
Source: ChemSusChem. 2025 Apr 23;18(12):e202500103. doi: 10.1002/cssc.202500103 (PMC12175045; doi:10.1002/cssc.202500103)
Supplement: Supplementary file 1 — Supplementary Material [file CSSC-18-e202500103-s001.pdf]

Supplementary Information

Solid-State NMR Investigations of the Lithium- and Sodium-Storage Mechanisms of Pyrolytic Phosphorus-Carbon Composites

Cassius Clark[a,c], Christopher A. O'Keefe[a], Dominic S. Wright[a,b] and Clare P. Grey*[a,b,c]

[a] Dr. C. Clark, Dr. C. A. O'Keefe, Prof. D. S. Wright, Prof. Dame. C. P. Grey
Yusuf Hamied Department of Chemistry
University of Cambridge
Lensfield Road, CB21EW, United Kingdom
E-mail: cpg27@cam.ac.uk

[b] Prof. D. S. Wright, Prof. Dame C. P. Grey
The Faraday Institution
Quad One, Harwell, Science and Innovation Campus, Didcot, United Kingdom

[c] Dr. C. Clark, Prof. Dame C. P. Grey
Cambridge Graphene Centre,
University of Cambridge,
9 JJ Thompson Avenue, Cambridge, CB30FA, United Kingdom

S1 – Post-Cycling SEM

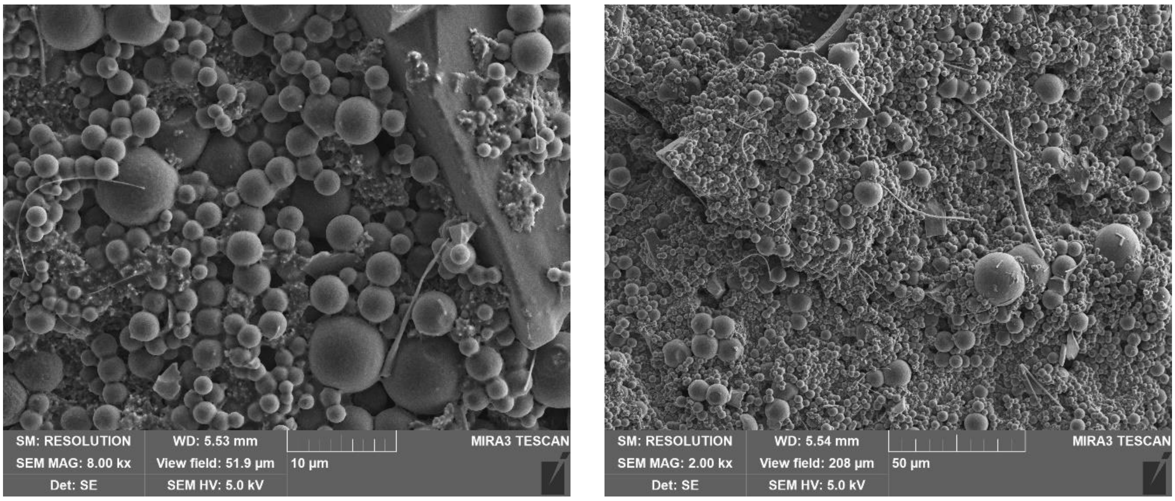

Figure S1.1. Post-cycling SEM analysis of PDC electrodes after 50 cycles in lithium half-cells. The spheres remain intact, coated in an SEI, with the presence of electrolyte, binder, and fiberglass separator visible.

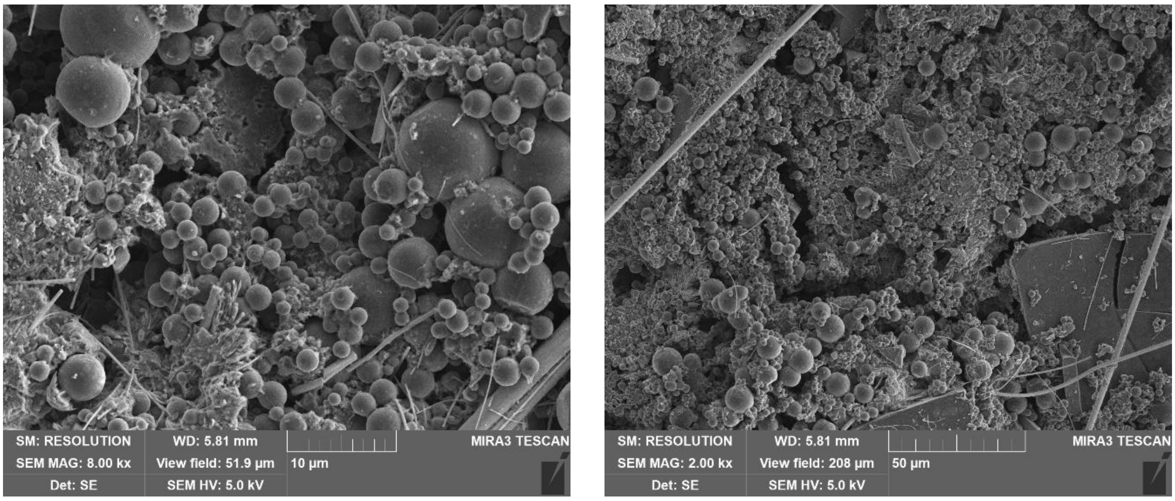

Figure S1.2. Post-cycling SEM analysis of PDC electrodes after 50 cycles in sodium half-cells. The spheres remain intact, coated in an SEI, with the presence of electrolyte, binder, and fiberglass separator visible.

S2 – X-ray Photoelectron Spectroscopy Depth Profiling

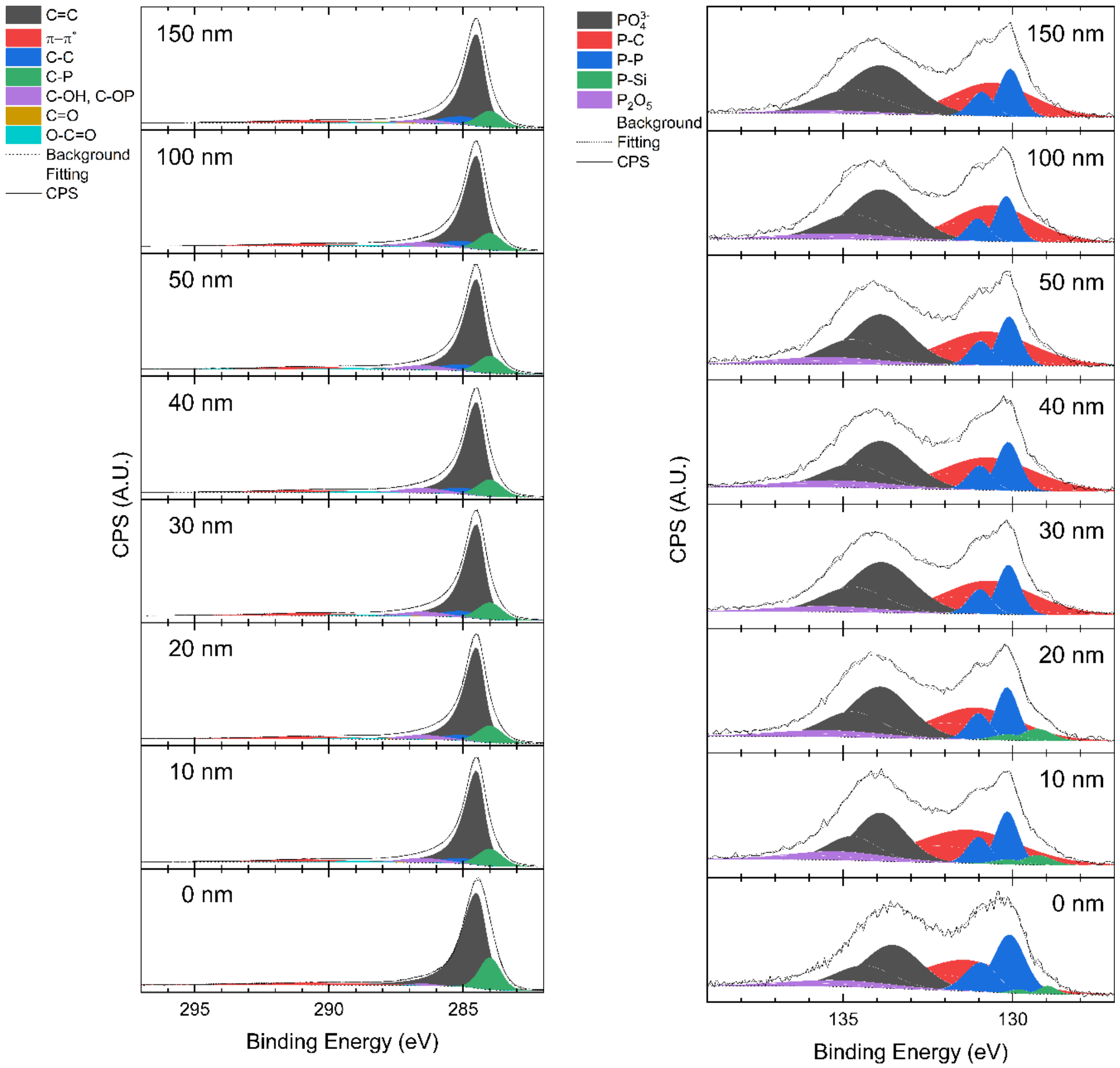

Figure S2.1. Peak fitting at measured approximate depths for PDCs with fitted peaks. Left: C1s region; Right: P2p region.

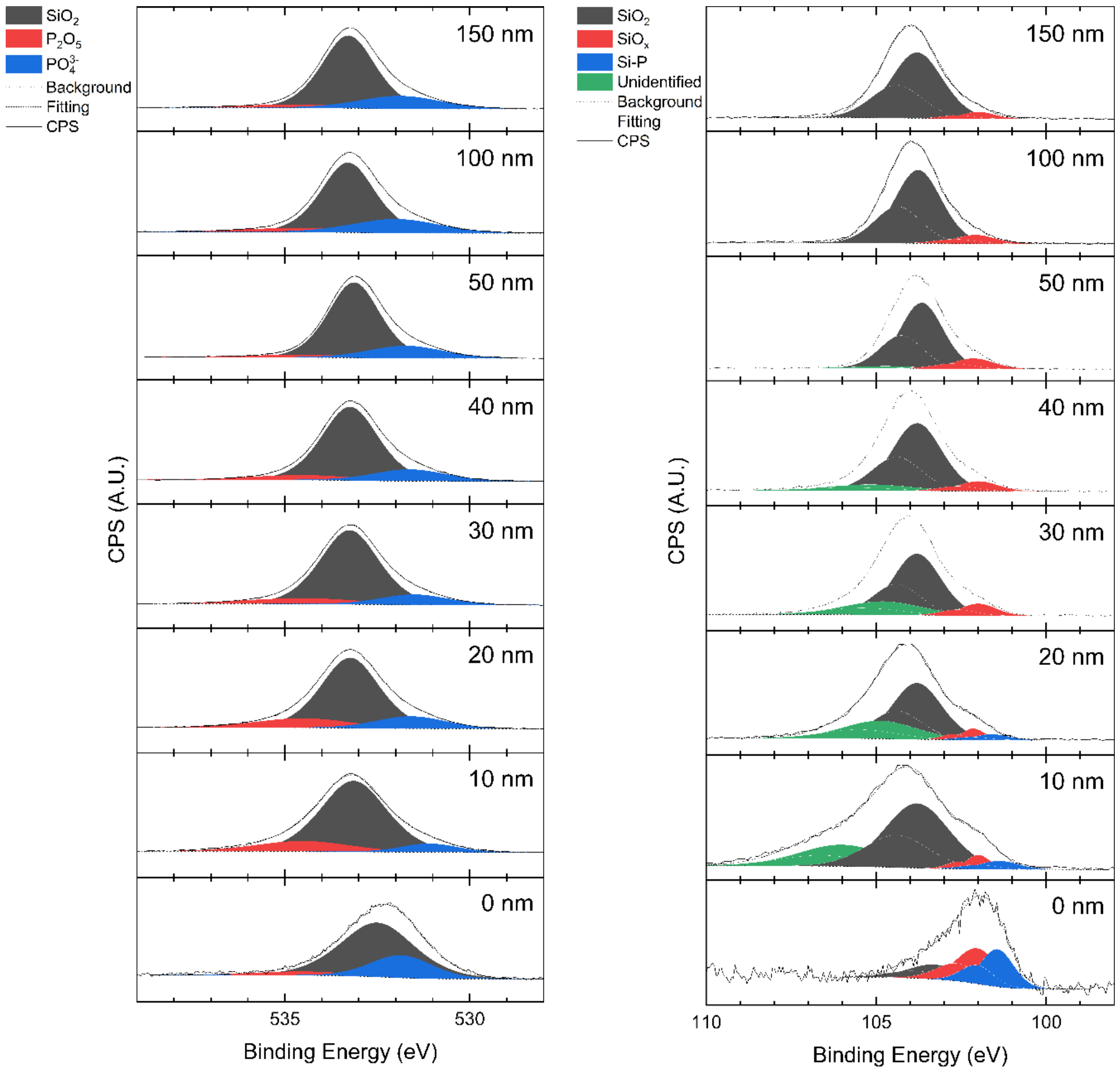

Figure S2.2. Peak fitting at measured approximate depths for PDCs with fitted peaks. Left: O1s region; Right: Si2p region.

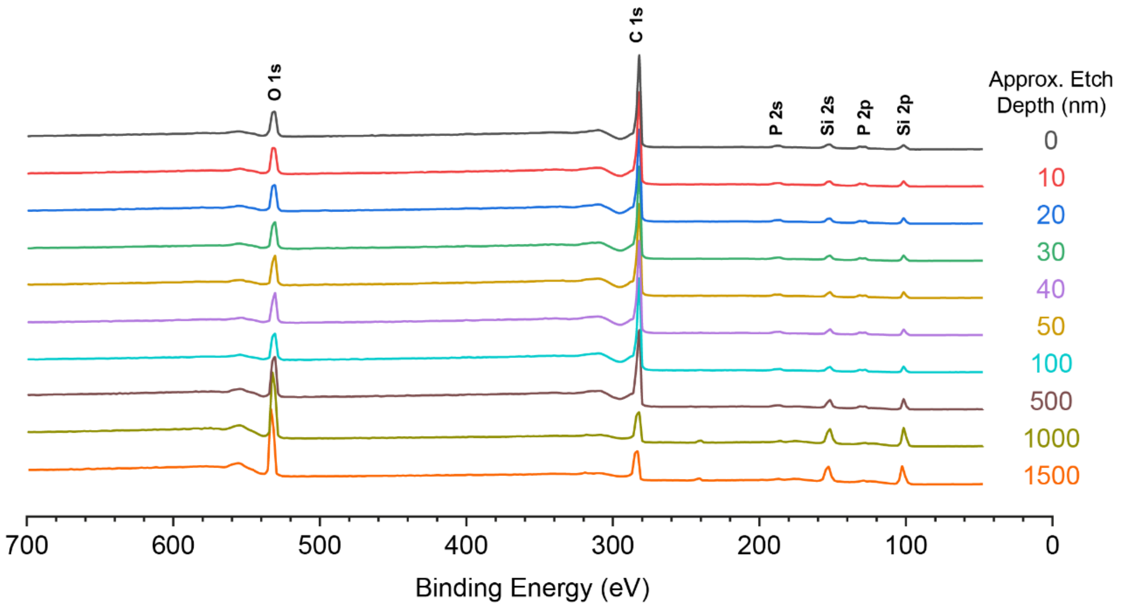

Figure S2.3. Survey scans from XPS measurements coupled with ion-etching for a PDC sample at varying approximate depths.

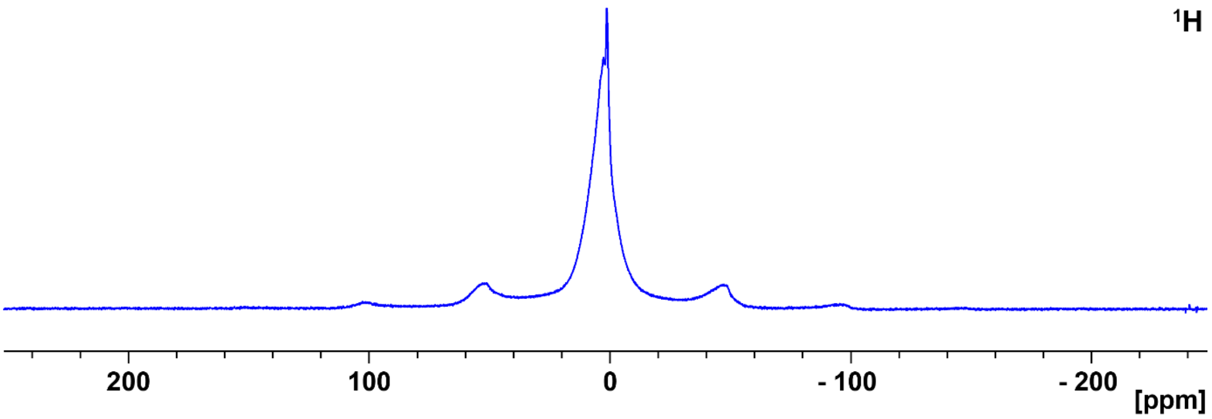

Figure S3. ^{1}H SSNMR spectrum of desodiated PDC material.

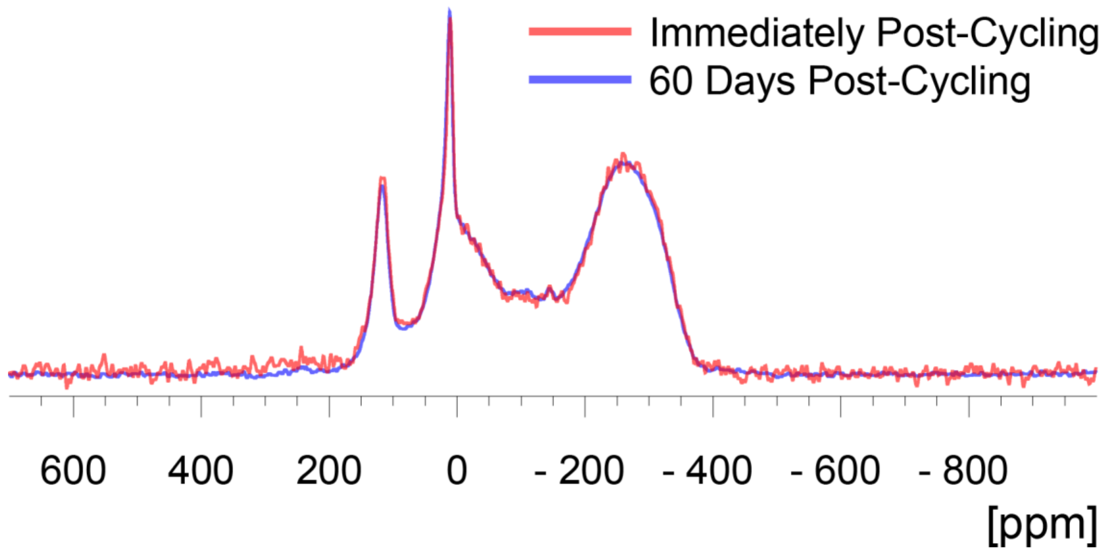

Figure S4. A comparison of the ^{31}P solid-state NMR spectra of PDC lithated at 0.005V taken immediately after cycling and another, using the same sample stored inside an air-tight rotor outside of a glovebox after 60 days. Minimal degradation between the samples is observed. Spinning speed, ν_{rot} = 25 kHz.

Symbol	Variable	EDS	SSNMR	EDS and SSNMR Calculations	XPS	XPS Calculation
m1	Total Assumed Mass (g)	1	1	Assumed	N/A	N/A
$m\%_C$	Mass % C	84.6	98.4	Experimental	87.9	$100 * m_C / (m_C + m_P)$
$m\%_P$	Mass % P	15.4	1.6	Experimental	12.1	$100 * m_P / (m_C + m_P)$
m_C	Mass C (g)	0.846	0.984	$(m_C * m1)/100$	1.87E-21	$mol_C * ma_C$
m_P	Mass P (g)	0.154	0.016	$(m\%_P * m1)/100$	2.57E-22	$mol_P * ma_P$
R_m	Mass Ratio	5.5	61.5	m_C/m_P	7.3	m_C/m_P
m	Total Mass (g)	1	1	$m_C + m_P$	2.12E-21	$m_C + m_P$
mol_C	mol C	7.05E-02	8.20E-02	m_C / ma_C	1.55E-22	at_C / N_A
mol_P	mol P	4.98E-03	5.18E-04	m_P / ma_P	8.30E-24	at_P / N_A
at_C	atoms C	4.25E+22	4.94E+22	$mol_C * N_A$	93.6	$1 * at\%_C$
at_P	atoms P	3.00E+21	3.12E+20	$mol_P * N_A$	5	$1 * at\%_P$
$at\%_C$	Atomic % C	93.40	99.37	$100 * at_C / (at_C + at_P)$	93.6	Experimental
$at\%_P$	Atomic % P	6.60	0.63	$100 * at_P / (at_C + at_P)$	5	Experimental
R_{at}	Atomic Ratio	14.2	158.4	at_C / at_P	18.7	at_C / at_P
C_C	Theoretical Capacity from C (mAh)	314.7	366.0	$m_C * TC_C$	6.94E-19	$m_C * TC_C$
C_P	Theoretical Capacity from P (mAh)	395.6	41.1	$m_P * TC_P$	6.59E-19	$m_P * TC_P$
C	Total Theoretical Capacity (mAh)	710.3	407.2	$C_C + C_P$	1.35E-18	$C_C + C_P$
SpC	Total Theoretical Specific Capacity (mAh/g)	710.3	407.2	C / m	637.7	C / m

Table ST1. Calculations of the theoretical capacity of PDC based on the mass ratios (determined through EDS and solid-state NMR) and atomic rations (determined through XPS) of carbon and phosphorus present. Calculations assume that the carbon present acts as graphitic carbon and that phosphorus undergoes complete conversion to Li_3P during lithiation, using the theoretical capacity values in Table ST2. Additional constant values in Table ST2 are also used in calculations.

Symbol	Constant	Value	Unit
ma_C	Atomic Mass C	12	u
ma_P	Atomic Mass P	30.91	u
N_A	Avogadro's Constant	6.02E+23	/mol
TC_C	Theoretical Capacity C	372	mAh/g
TC_P	Theoretical Capacity P	2569	mAh/g

Table ST2. Additional constants used for the calculations in table ST1.
